# Microstructure and Microhardness Evolution of Mg–8Al–1Zn Magnesium Alloy Processed by Differential Speed Rolling at Elevated Temperatures

**DOI:** 10.3390/ma17164072

**Published:** 2024-08-16

**Authors:** Saad A. Alsubaie, Ahmed S. J. Al-Zubaydi, Emad A. Hussein, Meshal Y. Alawadhi

**Affiliations:** 1 Department of Manufacturing Engineering Technology, College of Technological Studies, The Public Authority of Applied Education and Training (PAAET), P.O. Box 42325, Shuwaikh 70654, Kuwait; my.alawadhi@paaet.edu.kw; 2 School of Applied Sciences, University of Technology-Iraq, Baghdad 10001, Iraq; ahmed.s.alzubaydi@uotechnology.edu.iq; 3 School of Engineering, Faculty of Engineering and Physical Sciences, University of Southampton, Southampton SO17 1BJ, UK; 4 School of Production and Metallurgy Engineering, University of Technology-Iraq, Baghdad 10001, Iraq; emad.a.hussein@uotechnology.edu.iq

**Keywords:** differential speed rolling, magnesium alloy, shear punch test

## Abstract

Mg–8Al–1Zn magnesium alloy was successfully processed using deferential speed rolling (DSR) at temperatures of 400 and 450 °C for thickness reduction of 30, 50, and 70% with no significant grain growth and dynamic recrystallization. Using optical microscopy (OM), scanning electron microscopy (SEM), and transmission electron microscopy (TEM), the rolled microstructures were examined. Although the results indicate a slight reduction in grain size from the initial condition, the DSR processing of alloy at an elevated temperature was associated with a significant number of twins and a distribution of the fine particles of the second phase. The strength in terms of microhardness measurements and strain hardening in terms of shear punch testing was significantly improved in the rolled microstructure at room temperature. The existence of twins and widely distributed second-phase fine particles at twin boundaries reflected positively on the extent of the elongations in terms of shear displacements when microstructures were tested at elevated temperatures in the shear punch testing.

## 1. Introduction

In automotive applications, magnesium alloys are promising alternative lightweight metal alloys to replace denser ones like steel and aluminum due to their low density (1.74 gm/cm^3^) and their high specific strength (158 KN·m/kg). However, magnesium has an HCP structure, which illustrates poor workability and low ductility at room temperature due to its limited slip systems [1]. In HCP crystal structure materials, deformation at room temperature can be achieved by two dominant mechanisms: slip in the basal plane and/or mechanical twinning. To achieve homogenous deformation without cracks developing, five independent slip systems are required, according to von Mises’ criteria. Since materials that have HCP structure (such as magnesium alloys) have three slip systems in the basal plane (two of which are independent), this limited slip system resulted in brittleness and lack of ductility at room temperature, and these materials are considered to be hard-to-work materials [1,2]. Extruded Mg alloys have attracted attention because of their better mechanical properties than the cast Mg alloys [3]. The purpose of increasing the strength of Mg alloys is to compete with lightweight metal alloys and to expand its application in modern industry. For this to be achieved, further effort must be made. Even though the two popular techniques of severe plastic deformation (SPD), equal-channel angular pressing (ECAP) [4] and high-pressure torsion (HPT) [5], have become important in recent years because of their potential to produce bulk nanostructured materials [6], the previous processes are still on the laboratory scale and did not succeed in producing a large bulk material. Other techniques, such as rolling, have been developed to achieve grain size reduction by inducing intense plastic straining in a larger dimension material.

The differential speed rolling (DSR) process, which is one of the rolling subdivision types, can introduce a plastic strain at high values to metallic materials in comparison to traditional rolling within the cross-section of the deformed workpiece. This can be achieved by using two identical rotated rolls but at dissimilar speeds. The DSR process imposes shear and compression deformations simultaneously, leading to significant microstructural and properties alterations in the processed materials. The shear deformation is introduced by virtue of the differential speeds of rollers, whereas the compressive deformation is introduced by a reduction in the thickness of the processed material [7,8]. Advantages of the DSR processing of metallic materials are enhancement of both strength and ductility via bimodal grain refinement, applicability of large-scale parts, utilization of a lower number of rolling passes, and efficient deformity in comparison with conventional rolling by virtue of differential speeds and subsequent imposed shear deformation, leading to lower power consumption by the DSR facility in comparison with conventional rollers [9,10]. Some limitations come with the DSR process, such as increased friction and wear in the roller equipment due to the effect of differential speeds and the non-uniformity of deformation in the processed materials across thickness cross-section, leading to heterogeneity in the microstructure and properties [11,12]. However, this process can sometimes be considered continuous SPD processing due to its ability to improve strength and ductility at large-dimension manufacturing of worked pieces in comparison with HPT and ECAP [13,14]. 

The DSR process was used for the development of various metallic alloys based on Mg [15], Al [16], and Cu [17]. The outcomes in these aforementioned studies revealed an improvement in the mechanical properties of the deformed alloys due to intense imposed shear deformation and, consequently, grain refinement. Also, it was found that the rolling temperature and thickness reduction have an impact on the resultant microstructure and mechanical properties [18]. Wrought alloys are considered to display the lowest workability of magnesium alloys at room temperature yet have the greatest strength. In this category, the magnesium–aluminum system is the most important in alloys such as AZ31, AZ61, Mg–8Al–1Zn, and AZ91 [19,20,21,22]. It is well known that increasing the Al content in Mg alloys will increase the strength of the alloys. Although Mg–8Al–1Zn is a widely known magnesium alloy, there is still a lack of information on it as a material processed by different SPD processes [23]. In the current research, the DSR process will be employed to improve the properties of Mg–8Al–1Zn alloy sheets by examining the effect of processing temperatures on microstructural evolution and mechanical properties of the alloy, such as microhardness and shear punch properties of Mg–8Al–1Zn alloy samples.

## 2. Experimental Material and Procedure

**Figure 1:**
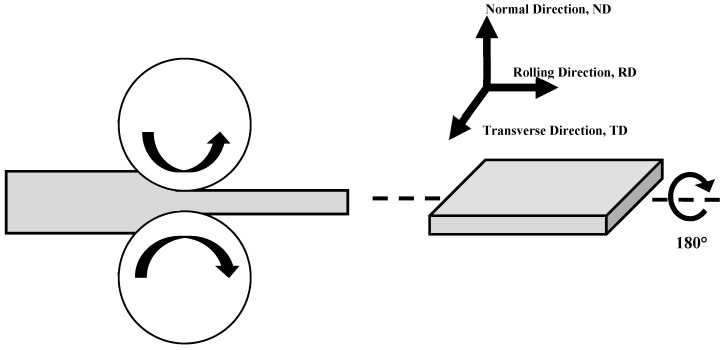
Schematic representation of the differential speed rolling process associated with sample rotation procedure between two successive passes [7].

The material used in this study was a commercial as-cast Mg–8Al–1Zn magnesium alloy (Mg–8.11% Al–0.45% Zn). This alloy came in the form of an extruded rod of 10 mm in diameter and 200 mm in length. The extrusion of the as-cast alloy was achieved at 300 °C using an extrusion ratio of 12; then, the resultant rod was solution heat treated at 400 °C for 12 h under an argon atmosphere followed by water quenching. This extruded alloy bar was cut into sheets for DSR processing with dimensions of 60 mm in length, 8 mm in width, and 2 mm in thickness. The average grain size and Vickers microhardness in the as-received extruded alloy bar were about 25 µm and 65 Hv, respectively. The sheet samples of Mg–8Al–1Zn alloy were rolled in DSR at 400 and 450 ± 10 °C, at reduction ratios of 30, 50, and 70%, corresponding to 1 pass, 2 passes, and 4 passes for each reduction ratio. Before the DSR process, the sheet samples were kept at the required DSR temperature for 5 min in the furnace and then rolled at the same temperature in order to keep the processing temperature during the DSR at the same level. The diameter ratio between the upper and lower rollers was 1.15, with rotation speeds of 2.5 m/min and 3 m/min for the upper and lower rollers. The DSR process was conducted along the length dimension of the samples (rolling direction, RD), which is aligned to the extrusion direction (ED) of the alloy bar. The final thickness was 1.4 mm by one pass (thickness reduction 10%), where each sample was rotated by 180° around the rolling direction (RD) between each two successive passes, as represented in Figure 1 [7]. The samples were reheated between each of the two successive passes, and the temperature of the rollers was maintained at the required DSR temperature using an embedded heating element inside the rollers, where the rolling was achieved under no lubrication condition. 

The Mg–8Al–1Zn sheet samples prior to and post-DSR process were mechanically ground and polished to a final mirror-like surface, then etched using acetic-glycol solution, rinsed with ethanol, and then air-dried. The microstructure was examined using optical microscopy (OM), scanning electron microscopy (SEM), and Transmission electron microscopy (TEM). X-ray diffraction analysis was also performed on alloy sheet samples to determine the presence of phases and calculate crystallite size and defect dislocations. Vicker microhardness measurements were achieved for sheet samples on normal direction surfaces of the sheets, and data were then plotted. The shear punch test (SPT) was conducted on alloy sheets at testing temperatures of 400 and 450 ± 10 °C using initial strain rates of 10^−1^, 10^−2^, and 10^−3^ s^−1^. The SPT samples were cut from the longitudinal direction of the DSR sheets with dimensions of 0.8 mm in thickness and 10 mm in diameter. A punch fixture with a cylindrical flat punch was used for shear application with a 3.175 mm diameter and 3.225 mm diameter of the receiving hole, as reported in [24]. The applied load in SPT was measured against the recorded punch displacement. All the data were acquired by a computer to determine the relation between the shear stress in the tested sheet samples and to draw load–displacement curves.

## 3. Results and Discussion

### 3.1. Microstructural Evolution

**Figure 2:**
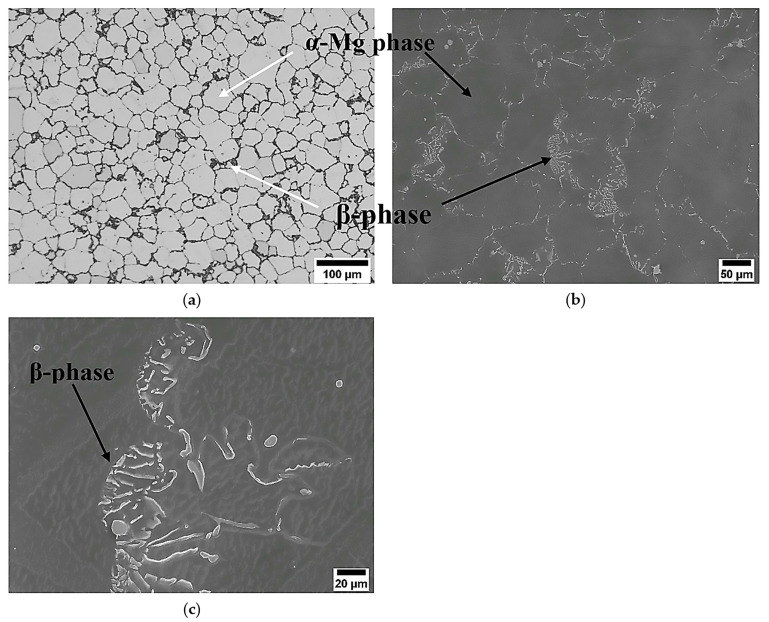
The microstructures of the as-received alloy as seen by OM in (**a**) and SEM in (**b**,**c**). The α-Mg matrix phase and β-phase are in white and dark appearances in (**a**), whereas these phases appear in SEM at reverse appearances to OM, as shown in (**b**). A magnified micrograph shows the morphology of β-phase in (**c**).

The initial microstructures of Mg–8Al–1Zn magnesium alloy prior to DSR processing are shown in Figure 2, where the average grain size of the microstructure was 25 µm, as seen by OM and SEM observations. These observations showed that the microstructure, prior to the process, consists of two main phases: the α-Mg phase, which forms the grain structure of the alloy (this phase appears as light and dark grey in OM and SEM micrographs, respectively) and the β-phase, which consists mainly of Mg_17_Al_12_, which decorates the grain boundaries of the α-Mg phase grains as seen in OM and SEM. This phase appears to have a black-and-white appearance in the aforementioned observations. A closer look at the microstructure shows that the β-phase appears as lamella and coarse particles at different areas around the grain boundaries. These phases were identified chemically using energy-dispersive spectroscopy (EDS) attached to the SEM unit, with the following ratios of (91.44 wt.% Mg–8.11 wt.% Al–0.45 wt.% Zn) for α-Mg phase microstructure and (55.2 wt.% Mg–44.3 wt.% Al–0.5 wt.% Zn) for β-phase microstructure.

**Figure 3:**
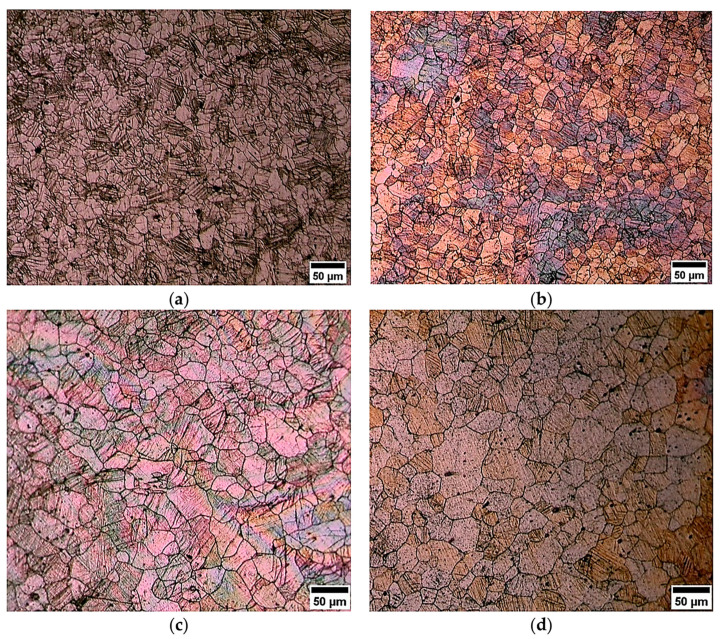
The DSRolled microstructures as seen by OM at a (**a**) rolling temperature of 400 °C for 30% of thickness reduction, (**b**) rolling temperature of 400 °C for 70% of thickness reduction, (**c**) rolling temperature of 450 °C for 30% of thickness reduction, and (**d**) rolling temperature of 450 °C for 70% of thickness reduction.

**Figure 4:**
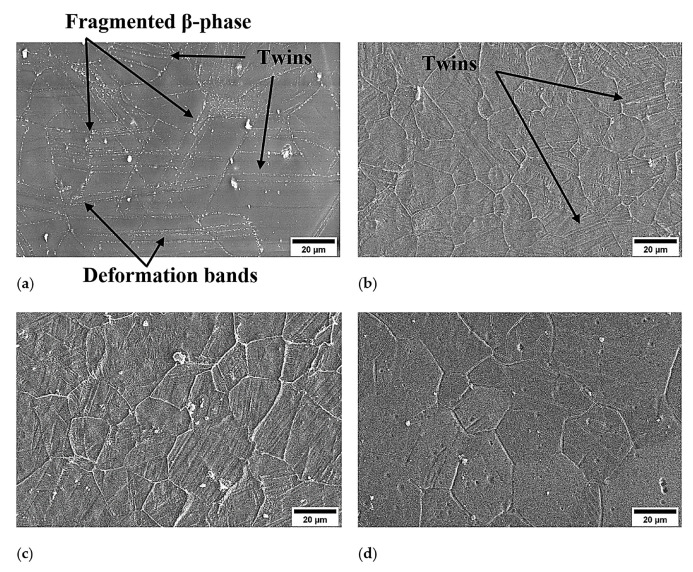
The DSRolled microstructures as seen by SEM at a (**a**) rolling temperature of 400 °C for 30% of thickness reduction, (**b**) rolling temperature of 400 °C for 70% of thickness reduction, (**c**) rolling temperature of 450 °C for 30% of thickness reduction, and (**d**) rolling temperature of 450 °C for 70% of thickness reduction.

**Figure 5:**
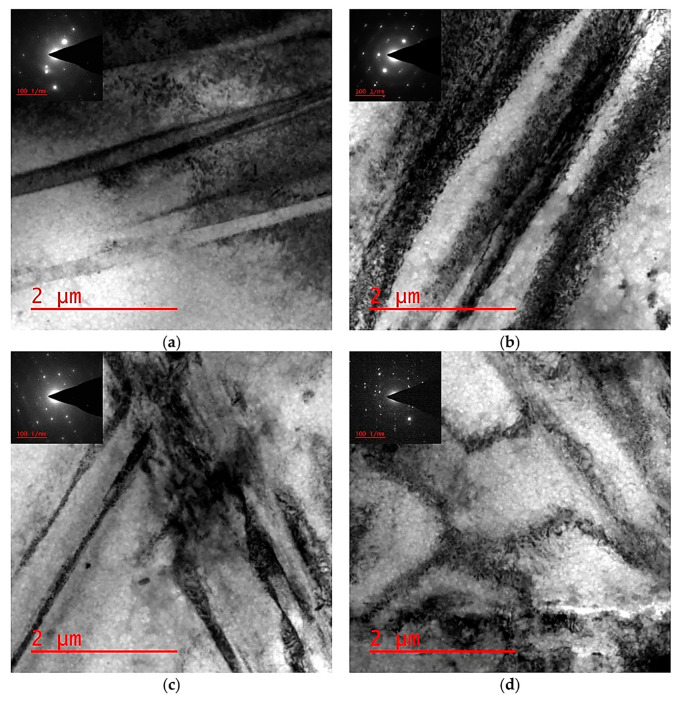
(**a**) Deformation bands, (**b**) nanotwins with dislocations, (**c**) intersected twins with dislocations, and (**d**) sub-grain formation surrounded with dislocations in the DSRolled alloy at a rolling temperature of 400 °C.

The processing of the alloy by DSR, starting from a thickness reduction ratio of 30%, 50%, and 70%, has been achieved successfully at elevated temperatures without surface cracking. The DSRolled microstructure showed initial grain refinement down to 15 and 20 µm for the samples rolled at 400 and 450 °C, respectively. The alloy at a rolling temperature of 400 °C showed extensive twinning and deformation bands as seen by OM observations in Figure 3a,b and SEM observations in Figure 4a,b, whereas less twinning and deformation bands were seen in DSRolled microstructures at a rolling temperature of 450 °C as seen by OM observations in Figure 3c,d and SEM observations in Figure 4c,d. The TEM observations of the DSRolled alloy at a rolling temperature of 400 °C are shown in Figure 5a–d, where the deformation bands of the nanoscale appeared clearly. Moreover, nanotwins associated with dislocations were also seen in the DSRolled alloys. These nanotwins were found to be intersected at some areas in the microstructure, resulting in the fragmentation of the grains down to nanoscales. This fragmentation has resulted in the formation of sub-grains of nanoscales surrounded by dislocations. A closer look at an area of dislocations shows that nanoparticles of β-phase were found due to fragmentation by virtue of imposed rolling strain in DSR processing. The β-phase in the rolled alloys at rolling temperatures of 400 and 450 °C has significantly fragmented into nanoparticles, especially at the lower reduction ratio of 30%. However, at higher reduction ratios up to 70%, this phase disappeared and seems to be dissolved within the α-Mg phase, as seen in SEM observations in Figure 4b,d. The disappearance of the β-phase was proportional to the increase in rolling temperature, as the effective dissolution of this phase happened at a rolling temperature of 450 °C rather than 400 °C, where it normally starts to dissolve at 400 °C as reported earlier due to the lower melting point of this phase in this alloy (460 °C). At a rolling temperature of 450 °C and at all ratios of reduction, the dissolution process happened at a faster rate, as seen in SEM observation in Figure 4c,d, which was associated with relatively grain growth and a small number of β-phase particles in the areas of dissolution [25,26]. However, the β-phase could not dissolve completely as each rolling pass took no more than one minute, whereas the complete dissolution process requires a time from 2 to 24 h [25,26,27,28]. In the initial stage of DSR deformation, i.e., at a reduction ratio of 30%, specifically at a rolling temperature of 400 °C, diffusional paths were provided for the solute atoms in the β-phase to diffuse within the α-Mg grains via introducing microstructural defects such as twins, dislocations and fragmentation of coarse particles which belonged to the β-phase. At an advanced stage of deformation, i.e., at a reduction ratio of 70% and a rolling temperature of 400 °C, a balance would appear between the dissolution and dynamic precipitation of the β-phase [25,29]. 

**Figure 6:**
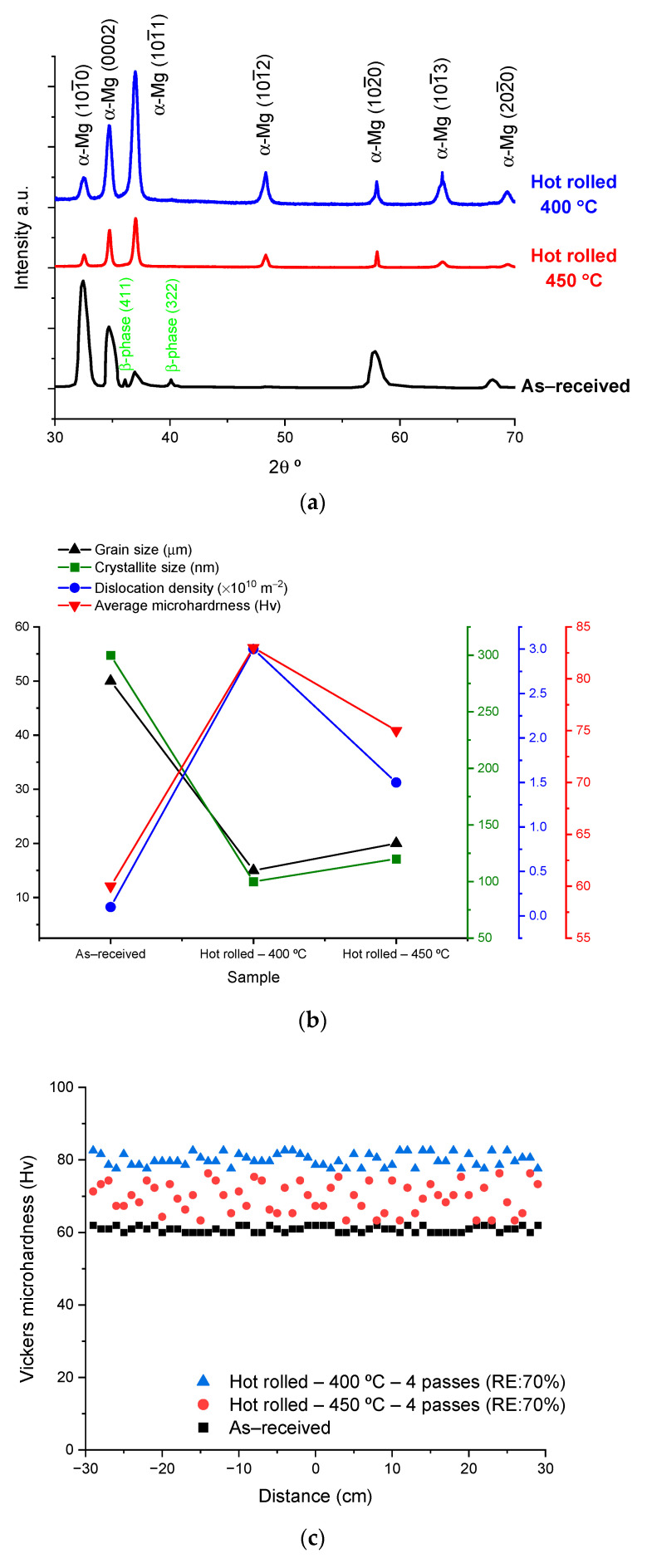

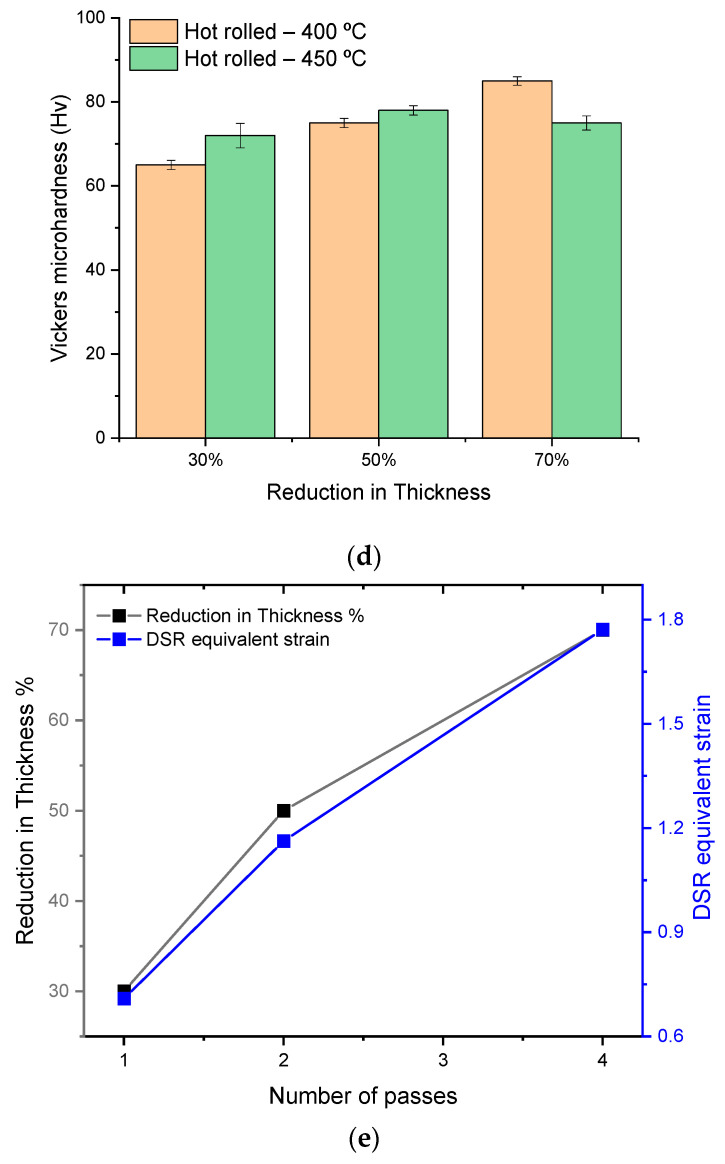
(**a**) XRD patterns for α-Mg phase and β-phase in the as-received and DSRolled alloys, (**b**) variation in grain size, crystallite size, dislocation density, and average microhardness in the as-received and DSRolled alloys, (**c**) variation in microhardness over the normal direction to the rolling direction for the as-received and DSRolled alloys for 4 passes (at 70% of reduction in thickness), (**d**) variation in microhardness with the reduction in thickness in as-received and DSRolled alloys, and (**e**) the total DSR equivalent strain imposed in the hot DSRolled alloys at each number of passes and ratio in thickness reduction.

The XRD patterns for the initial and rolled alloys are represented in Figure 6a, where the rolled alloys showed a clear tendency for twin generation as observed by the twin planes of ($10\bar{1}1$), ($10\bar{1}2$), and ($10\bar{1}3$) for α-Mg phase. This tendency for twinning generation in the α-Mg phase was higher in the rolled alloy at a rolling temperature of 400 °C rather than 450 °C, with the disappearance of the β-phase at these rolling temperatures as shown by XRD patterns. A reduction in the crystallite size was observed in the rolled alloys at both rolling temperatures in comparison with the as-received alloy, as represented in Figure 6b. However, the value of the crystallite size was slightly higher in the alloy rolled at a rolling temperature of 450 °C rather than 400 °C. The reduction in the crystallite size was associated with a slight increase in the defects in the rolled alloys at both rolling temperatures in comparison with the as-received alloy. The measurements of the grain and crystallite sizes were consistent for both as-received and rolled alloys.

**Figure 7:**
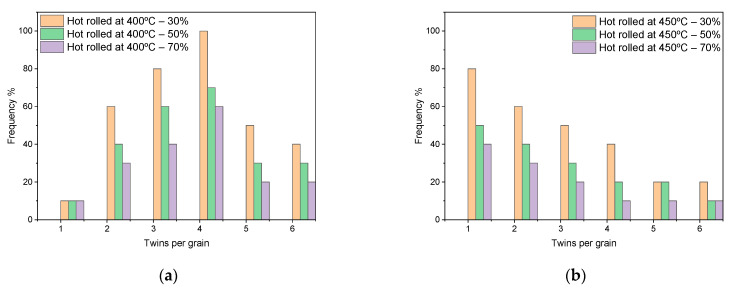
Twin distribution in the DSRolled alloy at rolling temperatures of (**a**) 400 °C and (**b**) 450 °C.

The materials with hexagonal-close packed crystal structures, such as magnesium and titanium alloys, have a limited number of independent slip systems, which makes twinning a vital deformation mechanism at high imposed strains and elevated temperatures [20]. Normally, the magnesium would be crystallographically orientated towards the basal system before the deformation processing by rolling because the system requires the lowest critical resolved shear stress in comparison to the counter values for non-basal systems, such as the twin pyramidal system. However, at high imposed strains and/or elevated temperatures, the critical resolved shear stress of the twin pyramidal system decreases. This will lead to the activation of additional systems to accommodate the deformation at the aforementioned conditions. The activation of pyramidal deformation systems can be noticed with the increase in processing temperature above 225 °C [30,31]. In the current research, the rolling temperatures were 400 and 450 °C, which induced effectively under DSR equivalent straining (see Figure 6e) to activate the considerable pyramidal systems as seen by twinning that appeared in OM, SEM and via XRD peaks and represented by twin distribution in Figure 7. The DSR equivalent strain (ε) was calculated according to the following equation [12]:

$$\varepsilon =\sqrt{\varepsilon_{r}^{2}+\frac{\gamma^{2}}{3}}=\sqrt{[\frac{2}{\sqrt{3}}\cdot \ln\left(\frac{1}{1-r}\right){]}^{2}+\frac{1}{3}[\frac{1}{\left(h_{0}+h\right)}\cdot R\cdot \cos^{-1}\left(1-\frac{h_{0}-h}{2R}\right)\left(1-\frac{v_{2}}{v_{1}}\right){]}^{2}},$$

where $\varepsilon_{r}$ and γ represent the compression and shear deformation components, respectively; r represents the reduction in thickness and calculated based on initial thickness $(h_{0})\;$and final thickness (h) as $r=1-\frac{h}{h_{0}}$, R represents the ratio of roll radii, and $v_{2}$ and $v_{1}$ are the high and low speeds of rollers, respectively. It is noted that this equation does not directly imply the effect of rolling temperature and generated temperature due to friction between the sample and rolls in the calculation of the imposed strain in DSR processing. However, the DSR at a relatively low-speed ratio (<1) would not impose any significant increase in the temperature, whereas at a high-speed ratio (>2), the increase in the temperature would be considerable [32].

The existence of compressive load components during straining processing via DSR processing also contributed to the activation of these deformation planes. As the thickness reduction per each pass was maintained at about 10%, preceding the deformation, the activation of contraction twinning will be dominated rather than extension twinning to accommodate the deformation homogeneity at higher strain levels and temperatures. This can be attributed to the decrease in the sample thickness, which leads to a decrease in the angle between the normal direction (ND) and the radial force at the edge of the rollers. The radial force has two components: the radial force in the normal direction (ND) and the other in the rolling direction (RD). With further thinning in the sample thickness, radial force in the normal direction will be higher than in the rolling direction, leading to the reorientation of grain structure in the Mg-based alloys towards the activation of additional deformation twinning systems at elevated temperatures [33,34]. It seems that the grain structure in the magnesium alloy that rolled at 400 and 450 °C was refined extensively by twinning fragmentation, where the twins were generated at these temperatures and then intersected under rolling strain deformation. Due to the limited active slip systems in magnesium and its alloys at ambient temperatures, thus the expected deformation to occur in these materials would be via the activation of additional deformation systems. The activation of such systems, such as pyramidal twining systems, requires elevated temperatures, as in the case of deformation conditions in the current investigation. As a result of this extensive activity of twin deformation, deformation bands and shear bands have formed here to dominate the deformation mechanisms under such rolling conditions where no dynamic recrystallization has appeared but grain growth instead that was resisted by the twin formation and intersection here [30,35]. It can be seen from the microstructural observation that the deformation bands have extended to form shear bands to generate a homogenous plastic deformation due to the imposed strain via DSR processing. The generated shear bands were seen over many grains in the rolled microstructures despite the occurrence of grain growth. These bands normally come with intense shear regions, leading to the deformation of localized regions associated with localized dislocation areas and second-phase fragmentation, as seen by TEM observations, where the twinning cannot accommodate the intense imposed straining with further deformation. The occurrence of shear bands appears to be in the orientation of propagation of twinning or intersecting them [36,37]. The shear bands were also found to generate via double twin areas in the rolled alloy, where these areas were considered as nucleation sources for the generation of the sheared material to accommodate the localized deformation at these regions and keep the deformation continuity under DSR straining rather than grain growth at such elevated temperatures of rolling processing [33,34]. Normally, the existence of shear bands during rolling processing at temperatures below 300 °C is considered as nucleation sites for dynamic recrystallized misstructure [38,39]. However, in the current investigation, the rolling temperatures were 400 and 450 °C, where no dynamic recrystallization was noticed, but the development of subgrains, as seen by TEM observation, were formed instead at such rolling temperatures. Thus, the domination deformation mechanism was not only the slip but mainly the twinning over the orientations of planes of ($10\bar{1}2$) and ($10\bar{1}3$) twinning as indicated by XRD data [30,40]. It should be noted that the relatively initial grain structure in the current alloy with a high amount of aluminum content would also support the activation of twinning in magnesium alloys. Thus, the grain refinement via twinning segmentation of initial grains in the rolled alloy has facilitated the plastic flow of the alloy under rolling conditions despite the localized regions of deformation due to shear banding [30,41].

### 3.2. Mechanical Behaviour Evolution

The rolled alloys showed an increase in strength in terms of Vickers microhardness, as shown in Figure 6c, in comparison with the as-received alloy. This increase in the microhardness was proportional to the increase in the number of passes or reduction in thickness in the rolled alloys, as represented in Figure 6d. However, the alloy rolled at a rolling temperature of 450 °C showed less proportionality with the number of passes of DSR in comparison with the alloy rolled at a rolling temperature of 400 °C. It is worth noting that the reduction ratios in thickness of 30, 50, and 70% correspond to the number of rolling passes of one, two, and four passes, which correspond to DSR equivalent strains of 0.71, 1.16, and 1.77, respectively, as represented in Figure 6e.

The development in strength with regard to the microhardness measurements in the rolled alloy depends on the evolution in the grain structure regarding the grain size, crystallite size, and the distribution of β-phase. The grain refinement, twin generation, and fragmentation in the rolled alloy resulted in a hardness increase by virtue of obstruction of the generated dislocation motion during plastic deformation generated by the indenter. Normally, the deformation of magnesium alloys at room temperature would imply the activity of basal slip systems at low strains and non-basal slip systems at high strains. The existence of twins in the hexagonal-closed packed magnesium alloys leads to an increase in strain hardening under the deformation process due to obstruction of the generated dislocation motion at the twin boundaries. Additionally, the accompanying reduction in the grain size due to the intersection of twins and their boundaries leads to the occurrence of the dynamic effect of Hall–Petch at these conditions [26,42]. This can be explained according to dislocation pile-ups at the twin boundaries that result in regions of high-stress concentration, leading to shearing of the generated twins via shearing bands that extend over many grains. Thus, the dislocation pile-ups in these sheared regions, as well as at twin boundaries and around the formed subgrains, will impose significant obstruction to slip deformation, leading to obvious strain hardening in the rolled alloy. However, the hardening in the rolled alloy at a rolling temperature of 450 °C was slightly lower in comparison with the rolled alloy at 400 °C due to the effect of softening that lowers the density of dislocations [25,30]. This softening arises from the intersection of generated twins that leads to the formation of dislocation-free new grains that nucleated within the twin bands and, at grain boundaries, that slide over each other, leading to a notable material flow [20,36]. As a matter of fact, the dynamically recrystallized new grains were hardly visible in this work since their formations need time and area, which were strongly restricted by very short processing times for each sample in the DSR, where the grain growth appears. However, this does not negate their potential contribution to the softening of the alloy [36,43].

Despite the fact that the alloy was rolled in DSR processing at elevated temperatures, the strength in terms of microhardness measurements was higher than the initial unrolled alloy, which reflects the effect of the DSR imposed strain on the microstructural development and property evolution. It was anticipated that by processing the alloy at elevated temperatures, the grain growth would introduce a detrimental effect on the performance of the hot rolled alloy, but the current results showed contrary to this view. The distribution of the β-phase would also introduce an effect of strain hardening in the hot rolled alloy. From SEM observation, this phase was in the form of brittle lamella and/or agglomerates in the unrolled initial alloy. In this case, the effect of this phase in obstructing the dislocation motions and accumulating these dislocations at interfaces of coarse grain/β-phase would have lower significance. However, the hot rolled alloys showed a finer β-phase with wide-spread distribution due to the nature of rolling processing. Thus, the fine particles of β-phase act as excellent obstructions to the dislocation activities at subgrain/fine grain/β-phase and twin boundaries, leading to additional contribution for the strain hardening at ambient temperatures [38,39].

**Figure 8:**
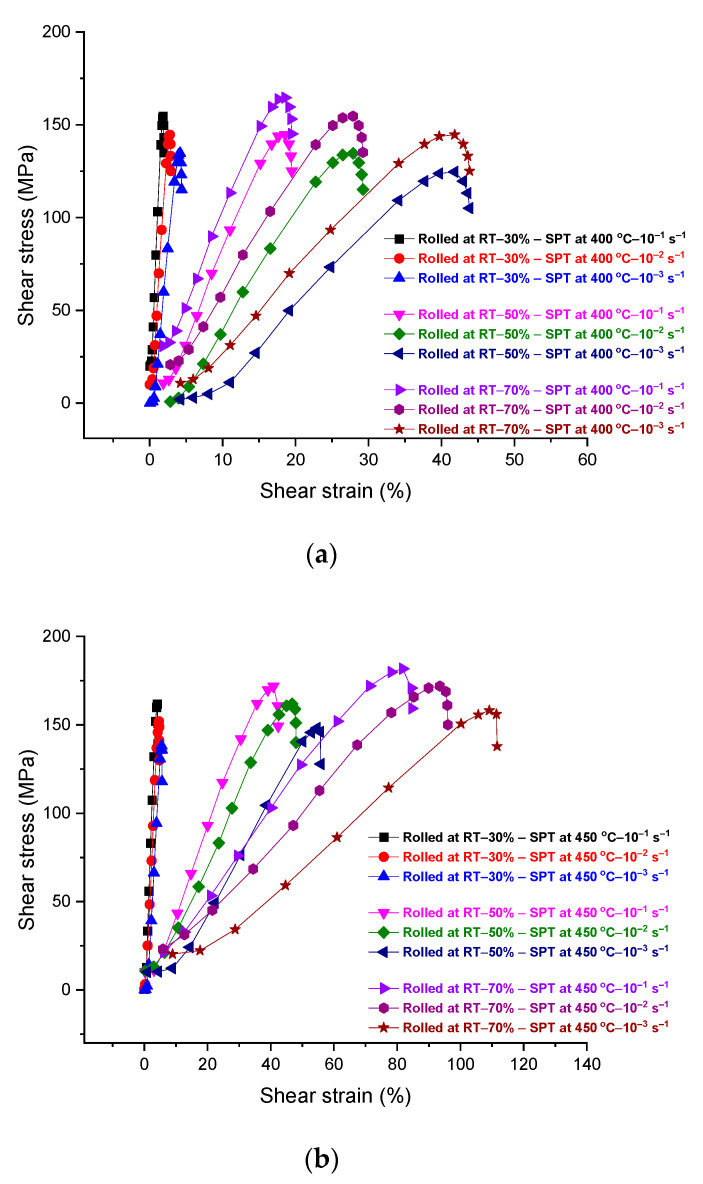
Shear stress–strain curves for the DSRolled alloy at room temperatures for different ratios of thickness reduction and then tested in shear punch testing at testing temperatures of (**a**) 400 °C and (**b**) 450 °C at different strain rates.

**Figure 9:**
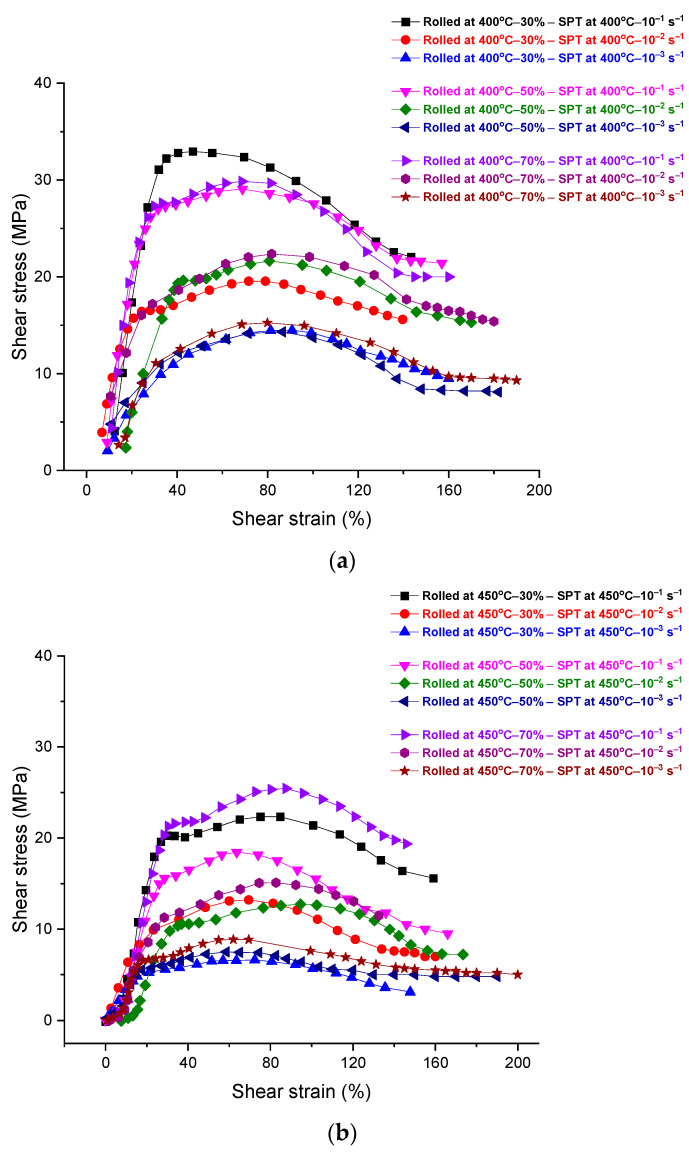
Shear stress–strain curves for the DSRolled alloy at rolling temperatures of 400 and 450 °C for different ratios of thickness reduction and then tested in shear punch testing at testing temperatures of (**a**) 400 °C and (**b**) 450 °C at different strain rates.

**Figure 10:**
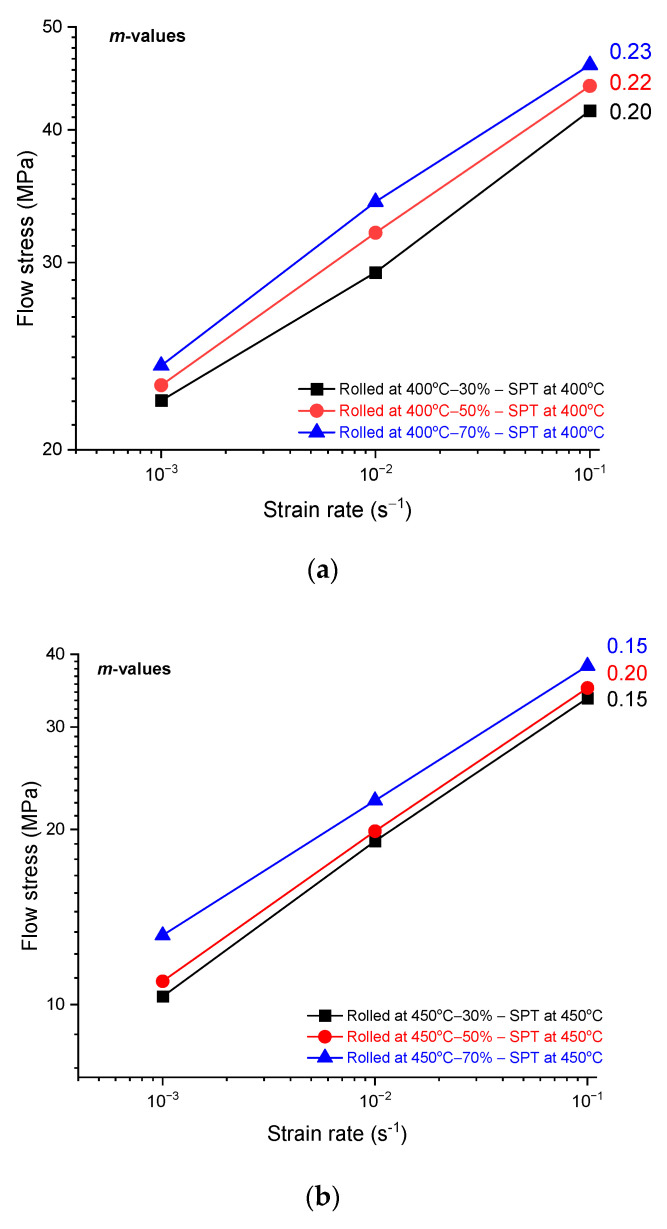
Values of strain rate sensitivity for the DSRolled alloy at rolling temperatures of 400 and 450 °C for different ratios of thickness reduction and then tested in shear punch testing at testing temperatures of (**a**) 400 °C and (**b**) 450 °C at different strain rates.

The mechanical behavior of the as-received and rolled alloys, as tested by shear punch testing, is represented by shear stress–strain plots in Figure 8 and Figure 9. The rolled as-received alloy at room temperature was tested using shear punch testing at testing temperatures of 400 and 450 °C, as shown in Figure 8. The samples of rolled as-received alloy showed high values of strain hardening at all rolling passes. A noticeable increase in the measured displacement is expressed by shear strain in the plots of shear stress–strain with increasing the number of rolling passes and testing temperature. However, the rolled as-received samples at 450 °C at all passes showed higher values of strain hardening and shear displacements with increasing the number of rolling passes, in comparison with the rolled as-received samples at 400 °C that approximately the same shear displacements at the same strain rates despite the number of rolling passes as in Figure 8. On the other hand, the alloy rolled at 400 and 450 °C and then tested in the shear punch testing at 400 and 450 °C showed considerable shear displacements, as in Figure 9, in comparison with the rolled as-received alloy at room temperature. These hot-rolled alloys showed higher values of strain hardening and lower shear displacements in an inverse manner with the increase in the strain rate and decrease in the number of rolling passes. However, the hot-rolled alloy at 400 °C showed a higher strain hardening than the hot-rolled alloy at 450 °C at all strain rates and a number of rolling passes. The values of strain rate sensitivity for the hot-rolled alloys after shear punch testing at 400 and 450 °C were calculated and represented in Figure 10. The aforementioned values were used to represent the deformation mechanism of the hot-rolled alloys at the testing temperatures. These values were slightly higher at a testing temperature of 400 °C rather than 450 °C. The positive value of strain rate sensitivity here reflects the increase in the shear strength with increasing strain rate at both testing temperatures.

In shear punch testing, the as-received samples that rolled at room temperature have presented a significant strain hardening in comparison to the hot rolled samples. This can be attributed to the strain hardening in the rolled alloy that increased significantly at room temperature as the number of passes increased or with the higher reduction in the thickness of sheet samples of the rolled alloy. In the rolled samples at room temperature, the level of defects during DSR processing is anticipated to reach higher levels in comparison with the hot-rolled samples, leading to a considerable obstruction to the tension deformation even at elevated testing temperatures that leads to high values of tensile strengths in the as-received samples post cold rolling. However, these samples showed moderate elongations in terms of shear displacements with slower strain rates and a higher number of passes. This can be explained in terms of grain refinement in the as-received samples that rolled at room temperature, which is expected to be much finer than for the hot-rolled samples. This would provide paths for grain-boundary sliding during the hot deformation in tensile testing at slower strain rates [24,44]. At DSR processing at room temperature, it is anticipated that the grain size reaches the ultrafine scale due to the high value of the defects that are imposed in the microstructure where no effects of dynamic recovery, recrystallization, or grain growth would appear. Thus, the achieved elongations in the as-received samples that were processed in DSR at room temperature were expected due to the formation of equiaxed and elongated microstructures towards the rolling direction, leading to an enhancement in the ductility. However, these elongations were even better in the hot-rolled samples rather than the cold-rolled counterparts, where the hot-rolled samples showed significant improvement in the achieved elongations in terms of shear displacements with increasing the number of passes. This can be attributed to the significant activity of twinning that was generated during hot rolling and then persisted during the hot deformation in the shear punch testing. The deformation of magnesium alloys at room temperature is limited mainly to the basal slip activity that leads to constraints in the material flow and elongations at ambient temperatures. However, at elevated temperature deformation, these alloys tend to initiate twins to accommodate the deformation following the twin-induced mechanism, associated with the increase in the slip deformation mechanism leading to the end in the formation of new dynamic recrystallized grains [45,46]. In the current investigation, the hot-rolled alloys showed no dynamic recrystallization at the range of processing temperatures but instead significant activity of the generated twins. Thus, the edge dislocations that accumulated and moved over the pyramidal twin system would be bound on the slip basal system, and their mobility would increase with increasing temperature, leading to a significant flow of the alloy under elevated temperature deformation. The values of strain rate sensitivity, as represented in Figure 10, and the distribution of twins data, as represented in Figure 7, confirm the domination of pyramidal cross-slip as a main mechanism of the alloy flow during shear punch testing at temperatures 400–450 °C [45,47]. The distribution of the fine particles for the β-phase after the hot rolling is expected to have an impact on the alloy flow and elongation under the condition of hot deformation in the current study. The β-phase has fragmented and refined down to the nanoscale and distributed along the twin boundaries and inside the grains, leading to strain hardening at the initial stages of hot deformation followed by the softening effect of the alloy under this condition. This can be attributed to the pinning effect of these fine particles to the motion of dislocation and material flow at the early stage of deformation despite the elevated temperature of the test. These particles then act as a lubricant, which facilitates the glide of grains over grains and twin boundaries at testing temperatures of 400–450 °C (673–723 K); this testing temperature represents about (0.91–0.98) of the melting point of this phase of 460 °C (733 K) [48,49].

## 4. Conclusions

1. A commercial Mg–8Al–1Zn magnesium alloy was successfully processed by differential speed rolling at elevated temperatures of 400 and 450 °C without any significant grain growth at these rolling temperatures for a reduction percentage of 30% and 70%.
2. Considerable twinning was observed in DSRolled alloy with no grain growth nor dynamic recrystallization indicating that twin deformation is the dominant deformation mechanism in addition to slip mechanism.
3. The DSRolled alloy showed significant elongation in terms of shear displacements at testing temperatures of 400 and 450 °C.
4. The existence of twins and the distribution of fine particles of β-phase (Mg_17_Al_12_) played important roles in the behavior of DSRolled alloy under the hot shear deformation. These two factors inhibit the significant grain growth, improve the strain hardening and improve the alloy flow under the hot deformation conditions.

## Data Availability

The original contributions presented in the study are included in the article, further inquiries can be directed to the corresponding author.
